# Postoperative Mortality and Associated Risk Factors in Emergency General Surgery: A Single-Center Cohort Study From Greece

**DOI:** 10.7759/cureus.117656

**Published:** 2026-10-08

**Authors:** Alexis Terras, Myrto D Keramida, Annita Loizou, Ifaistion M Palios, Panagiotis Dorovinis, Nikolaos Machairas, Stylianos Kykalos, Dimitrios Dimitroulis, Michael Samarkos, Nikolaos I Nikiteas, Gerasimos Tsourouflis

**Affiliations:** 1 2nd Department of Propaedeutic Surgery, Laiko General Hospital, Athens Medical School, National and Kapodistrian University of Athens, Athens, GRC; 2 1st Department of Propaedeutic and Internal Medicine, Laiko General Hospital, Athens Medical School, National and Kapodistrian University of Athens, Athens, GRC

**Keywords:** acute care surgery, emergency general surgery, emergency laparotomy, greece, nela, postoperative mortality, risk factors

## Abstract

Introduction

Emergency General Surgery (EGS) comprises a heterogeneous group of acute surgical conditions that require prompt evaluation and, frequently, urgent operative intervention. Patients presenting for EGS often have significant comorbidity, physiological derangement, sepsis, or hemodynamic instability, and are exposed to substantially higher postoperative morbidity and mortality than patients undergoing elective surgery. Although contemporary Greek data on emergency laparotomy have recently emerged, outcomes across the broader operatively managed spectrum of EGS remain ill-determined. We aimed to characterize 30-day postoperative mortality and associated risk factors across operatively managed EGS - defined as procedures of immediate or urgent priority - in a single Greek tertiary center.

Methods

We conducted an ambispective, single-center observational cohort study of adults undergoing emergency general surgery of National Confidential Enquiry into Patient Outcome and Death (NCEPOD) immediate or urgent priority at a tertiary academic center in Athens, Greece, between January 2020 and July 2025. The primary outcome was 30-day postoperative mortality. Associations with mortality were examined using univariable logistic regression and a multivariable model incorporating age, American Society of Anesthesiologists (ASA) grade, preoperative sepsis severity, emergency laparotomy, and generalized peritonitis/gross contamination. Established emergency-surgery risk-prediction scores (National Emergency Laparotomy Audit (NELA), Emergency Surgery Acuity Score (ESAS), Portsmouth Physiological and Operative Severity Score for the enUmeration of Mortality and morbidity (P-POSSUM), and Surgical Risk Scale (SRS) were additionally evaluated for discrimination in an exploratory analysis.

Results

Of 2,506 screened cases, 2,040 patients were included, with a 30-day mortality of 7.6% (156/2,040), increasing to 12.5% (52/415) after emergency laparotomy and 11.6% (42/361) among NELA-eligible patients. Mortality varied markedly by diagnosis (0.7% (3/430) for appendicitis to 50.9% (29/57) for bowel ischemia) and increased progressively with ASA grade (2.0% (8/403) for ASA I to 52.9% (9/17) for ASA V) and preoperative sepsis severity (3.6% (41/1141) without sepsis to 45.6% (31/68) with septic shock). In the multivariable model (n=2,019), mortality was independently associated with ASA grade (ASA III vs I: aOR 2.53, 95% CI 1.05-6.08; ASA IV-V vs I: aOR 4.53, 95% CI 1.67-12.30) and preoperative sepsis severity (systemic inflammatory response syndrome (SIRS)/infection vs none: aOR 1.95, 95% CI 1.26-3.03; sepsis or septic shock vs none: aOR 3.52, 95% CI 2.10-5.88). Risk-prediction scores showed moderate discrimination on exploratory analysis (AUROC 0.66-0.72); calibration was not assessed.

Conclusions

Thirty-day mortality following emergency general surgery was substantial and varied markedly according to underlying diagnosis, ASA grade, and preoperative sepsis severity. In this multivariable model, ASA grade and sepsis severity were independently associated with mortality although these associations were sensitive to model specification and residual confounding by diagnosis cannot be excluded. Established risk-prediction scores showed only moderate discrimination in this heterogeneous EGS population, supporting the need for dedicated validation and calibration before their use for risk prediction or benchmarking in Greek practice.

## Introduction

Emergency General Surgery (EGS) encompasses a heterogeneous spectrum of acute surgical conditions, including acute appendicitis, acute cholecystitis, incarcerated or strangulated hernia, bowel obstruction, perforated hollow viscus, peritonitis, mesenteric or bowel ischemia, anorectal sepsis, necrotizing soft-tissue infection, and emergency oncological presentations, which require prompt surgical assessment and, in a substantial proportion, urgent operative intervention [1,2]. Unlike elective surgical practice, EGS patients frequently present with pre-existing comorbidity, physiological derangement, sepsis, hemodynamic instability, or the need for rapid, high-stakes therapeutic decision-making, factors that are well documented to increase postoperative morbidity and mortality [2-6].

Large administrative-database studies from the United States and national audits from the United Kingdom have consistently shown that EGS carries a disproportionately high burden of postoperative death and complications relative to its elective counterpart, with mortality rates ranging from approximately 4-8% in unselected EGS cohorts to 10-15% or more among patients requiring emergency laparotomy [4,5,7-9]. National quality-improvement initiatives, such as the UK National Emergency Laparotomy Audit (NELA), have highlighted wide variation in outcomes and prompted systematic risk stratification and structured perioperative pathways for this population [8]. Notably, a stepped-wedge cluster-randomized trial of one such national quality-improvement program (EPOCH) found no significant difference in 90-day mortality despite successful implementation of the care pathway, underscoring that improving outcomes in this population is not straightforward and that risk stratification alone is unlikely to be sufficient [10]. A range of risk-prediction tools has been developed to support risk communication and resource allocation in this population, including the American Society of Anesthesiologists (ASA) physical status classification [11]; the Portsmouth Physiological and Operative Severity Score for the enUmeration of Mortality and morbidity (P-POSSUM), itself a recalibration of the original POSSUM score [12,13]; the Surgical Risk Scale (SRS) [14]; the Emergency Surgery Acuity Score (ESAS) [15]; the subsequently derived Emergency Surgery Score (ESS), a distinct instrument with a different variable set [16]; and the NELA risk model [8]. ESAS and ESS are frequently conflated in the literature but are separate scores and are not interchangeable.

Contemporary Greek data addressing this burden have begun to emerge only recently, and almost exclusively in the context of emergency laparotomy. The Hellenic Emergency Laparotomy Study (HELAS), a prospective multicenter cohort of 633 patients across 11 Greek and Cypriot hospitals, reported a 30-day mortality of 16.3% after emergency laparotomy and identified age, ASA grade, dependent functional status, ascites, sepsis severity, and diabetes as independent predictors of death; notably, postoperative critical care utilization was substantially lower than in the UK NELA cohort (25.8% vs. 56.8%), a difference the authors proposed as a partial explanation for the comparatively higher Greek mortality [17]. Building on the same cohort, a subsequent study formally externally validated four mortality-prediction tools - ACS-NSQIP, NELA, P-POSSUM, and POTTER - in Greek emergency laparotomy patients: all four showed good discrimination (concordance statistics 0.79-0.85), but all except a surgeon-adjusted ACS-NSQIP model were poorly calibrated [18]. A related analysis of the same cohort separately developed and internally validated a prediction model for serious postoperative complications after emergency laparotomy [19]. The Emergency Surgery Score (ESS) has separately been prospectively validated in a Greek bi-institutional cohort [20]; we are not aware of any equivalent evaluation of ESAS, SRS, or P-POSSUM in a Greek emergency general surgery population.

This emerging body of work has substantially advanced the characterization of emergency laparotomy outcomes and risk-prediction tool performance in Greece. However, outcomes across the broader and more heterogeneous spectrum of emergency general surgery beyond laparotomy remain less well-described, and the risk-prediction instruments evaluated in Greece to date have been assessed almost entirely within laparotomy cohorts. In particular, ESAS, which is distinct from the ESS validated by Christou et al. [20], has, to our knowledge, not previously been examined in Greek patients, and neither SRS nor P-POSSUM has been assessed across an unselected, operatively managed EGS population in Greece.

The present study therefore aimed to characterize 30-day postoperative mortality across the spectrum of operatively managed emergency general surgery at a Greek tertiary center and to identify patient-, disease-, and procedure-related factors associated with mortality. We additionally explored the discriminatory performance of established EGS risk-prediction tools as a secondary, exploratory analysis; calibration was not assessed, and these findings are not intended to guide bedside use of the scores.

## Materials and methods

Study design and setting

This ambispective, single-center, observational cohort study was conducted at the 2nd Department of Propaedeutic Surgery, “LAIKO” General Hospital of Athens, a tertiary academic surgical department affiliated with the National and Kapodistrian University of Athens.

Ethical approval

The study was approved by the Scientific Council of “LAIKO” General Hospital on 31 May 2023 (Protocol number: 354). The approval covered both a retrospective review of records for patients operated on between January 2020 and the date of approval, and prospective data collection for the remainder of the study period; the study is therefore ambispective in design, comprising a retrospective phase (January 2020 to May 2023) and a prospective phase (June 2023 to July 2025). Data were extracted from departmental electronic clinical records and entered into a coded, anonymized electronic database without direct patient identifiers. All data handling adhered to the principles of medical confidentiality, patient anonymity, and applicable personal data protection regulations. The study was conducted in accordance with the Declaration of Helsinki and with Regulation (EU) 2016/679 (General Data Protection Regulation); the anonymization key was held separately by the investigators and no directly identifying data left the institution.

Study period and population

The study population comprised adult patients who underwent an emergency general surgical operation at the study department between January 2020 and July 2025. Eligible index conditions included acute appendicitis, acute cholecystitis and other acute biliary disease, incarcerated or strangulated hernia, ileus or bowel obstruction, perforation of a hollow viscus, peritonitis, bowel ischemia, anorectal abscess and other perianal sepsis, necrotizing soft-tissue infection, and emergency presentations of colorectal or other oncological disease.

For the purposes of this study, an emergency operation was defined as an unscheduled procedure performed during the index admission and classified as National Confidential Enquiry into Patient Outcome and Death (NCEPOD) urgency class "immediate" or "urgent". Urgency was assigned according to the designation documented by the responsible surgical team at the time the patient was listed for theater - recorded prospectively during the prospective phase of the study, and abstracted from the operating theatre record and the admitting team's contemporaneous documentation during the retrospective phase. Operations documented as having been deliberately deferred beyond the index decision - for a trial of non-operative management, for further physiological optimization or investigation, or for scheduling convenience - were classified as NCEPOD "expedited", and were designated semi-urgent and excluded (see Exclusion criteria), as were operations that could have been scheduled electively. The NCEPOD classification defines the urgent category with reference to an intended interval of less than 24 hours from the decision to operate. In this study, urgency was assigned from the documented clinical designation at listing rather than from the interval subsequently achieved because the achieved decision-to-operation interval reflects theater availability, competing emergency workload, and out-of-hours staffing in addition to clinical intent, and may therefore exceed the intended interval in cases that were unambiguously urgent. The term "emergency" is used consistently throughout this manuscript to refer to operations meeting this definition.

Each of the 2,040 patients in the final cohort contributed a single eligible emergency episode. Where a patient underwent more than one emergency operation during the study period, only the first eligible episode was retained for analysis, so that each patient is represented exactly once and the observations analysed are independent. Where more than one acute surgical diagnosis was present at the time of operation, the diagnosis judged most directly responsible for the need for emergency surgery, or most clinically severe if this was ambiguous, was recorded as the primary diagnosis for classification purposes.

Inclusion criteria

Patients were included if they met all of the following criteria: age ≥18 years; underwent an emergency general surgical operation meeting the definition above; operation performed within the study period (January 2020-July 2025); availability of the core perioperative dataset; and a documented 30-day postoperative outcome.

The core perioperative dataset was defined a priori as the minimum set of fields required to characterize the patient, the operation, and the outcome, and comprised age and sex; ASA grade; the primary acute surgical diagnosis; the procedure performed and operative approach; NCEPOD urgency designation; admission; and 30-day vital status. Comorbidity and functional data required for the multivariable model - Charlson Comorbidity Index, Clinical Frailty Scale, and individual comorbid conditions - were likewise required. Cases in which any of these fields could not be established from the clinical record were excluded as having incomplete core data or missing 30-day outcome, as applicable.

Exclusion criteria

Patients were excluded if they were younger than 18 years, if the index condition fell outside the scope of general surgery, if the admission or operation was semi-urgent rather than an emergency, per the definitions above, if the procedure was a non-index operation or a reoperation, or if the 30-day postoperative outcome could not be ascertained. In accordance with the original study protocol, reoperations performed for complications of prior planned or elective general surgical procedures were specifically excluded, since these represent a distinct clinical population with a different risk profile from primary emergency presentations.

Patients undergoing surgery for traumatic injury, whether blunt or penetrating, were excluded, as were operations performed primarily for the management of traumatic visceral, vascular, or soft-tissue injury identified at laparotomy. Although emergency general surgery and trauma surgery are both encompassed by the wider discipline of acute care surgery, they constitute distinct populations: trauma patients are typically younger, carry a lower burden of chronic comorbidity, and sustain mortality that is driven predominantly by injury severity, hemorrhage, and mechanism rather than by physiological reserve and the septic sequelae of intra-abdominal pathology. Their outcomes are conventionally risk-adjusted using injury-specific instruments such as the Injury Severity Score [21] and TRISS [22], rather than the general surgical tools evaluated here.

Data source and variables

Data were collected by four researchers using a standardized data-collection proforma. Data for patients operated on between January 2020 and May 2023 were collected retrospectively from electronic clinical records, whereas from June 2023 onwards, data were recorded prospectively during the index admission. Missing or incomplete data were supplemented, where available, through structured review of the electronic clinical record. Recorded variables comprised demographic and preoperative, operative, postoperative, and risk-prediction data.

Demographic and preoperative data

Demographic and preoperative data included age, sex, body mass index, ASA physical status grade, Charlson Comorbidity Index, Clinical Frailty Scale score, functional dependence, comorbidities, chronic anticoagulant or antiplatelet therapy, chronic steroid use, primary diagnosis, preoperative laboratory parameters, sepsis category, vasopressor requirement, and preoperative mechanical ventilation. Recorded comorbidities included chronic obstructive pulmonary disease, chronic kidney disease, ischemic heart disease/heart failure, atrial fibrillation, diabetes mellitus, hypertension, and disseminated malignancy. Laboratory parameters included hemoglobin, white cell count, platelet count, C-reactive protein, urea, creatinine, albumin, sodium, international normalized ratio, and lactate.

Preoperative sepsis

Preoperative sepsis severity was classified as none, systemic inflammatory response syndrome (SIRS)/infection, sepsis, or septic shock according to the worst physiological status documented within 24 hours before surgery. Sepsis was defined, consistent with the Sepsis-3 framework, as suspected or confirmed infection with acute organ dysfunction attributable to it, identified from the treating team's contemporaneous assessment. Septic shock was assigned from contemporaneous documentation of sepsis with circulatory compromise requiring hemodynamic support. Concordance with the individually recorded fields was incomplete: of 68 patients so classified, 40 had a recorded vasopressor requirement, and 58 had a lactate greater than 2 mmol/L, with 37 meeting both. The 28 without a recorded vasopressor requirement had comparable acuity to those with one (21 with lactate >2 mmol/L, 17 requiring critical care, 30-day mortality 46.4% vs 45.0%). The sepsis categories should therefore be read as a pragmatic clinical severity gradient rather than a strict Sepsis-3 phenotype, and this is acknowledged as a limitation.

Operative data

Operative data included primary diagnosis, procedure type, surgical approach (open, laparoscopic, converted to open, or local/minor procedure), stoma formation, and intraoperative findings, including generalized peritonitis/gross contamination, fecal contamination, and bowel ischemia or necrosis. Emergency laparotomy was defined as a major gastrointestinal procedure - exploratory laparotomy or washout, colonic resection, small bowel resection, or stoma formation - performed via an open or laparoscopic-converted-to-open approach. The study was designed hierarchically: NELA procedural and indication criteria were then applied within this emergency laparotomy subgroup. The resulting NELA-eligible emergency laparotomy subgroup is therefore narrower than the full NELA case definition, which additionally includes procedures completed laparoscopically.

Postoperative data

Postoperative data included complications according to the Clavien-Dindo classification, postoperative sepsis, ICU admission and length of stay, reoperation and readmission within 30 days, total hospital length of stay, in-hospital mortality, and 30-day postoperative mortality. Complications were classified according to the highest Clavien-Dindo grade recorded for each patient. Planned postoperative critical care admission, routine postoperative investigations, and manifestations of the presenting surgical disease were not considered postoperative complications.

Risk-prediction scores

Four risk-prediction scores were calculated where the required input variables were available. The NELA predicted-mortality model was applied to the emergency-laparotomy subgroup, whereas P-POSSUM, ESAS, and SRS were calculated across the overall EGS cohort. P-POSSUM predicted mortality was derived from the physiological and operative severity scores using the Prytherch equation [12,13].

Study endpoints

The primary endpoint was 30-day postoperative mortality. Vital status at 30 days was ascertained hierarchically from the electronic health record, the national *AMKA*-linked health information system (*AMKA: Aριθμός Μητρώου Κοινωνικής Ασφάλισης* or social security registration number), or, when necessary, structured telephone follow-up with the patient or next of kin. Patients whose 30-day vital status could not be confirmed were excluded from the final analysis (n=20). Secondary endpoints included in-hospital mortality, postoperative complications, major complications (Clavien-Dindo grade ≥III), postoperative sepsis, ICU admission, reoperation and readmission within 30 days, and hospital length of stay.

Statistical analysis

Continuous variables were assessed for distribution and are presented as mean ± standard deviation or median (interquartile range), with comparisons performed using Welch’s t-test or the Mann-Whitney U test, as appropriate. Categorical variables are presented as number (percentage) and were compared using the chi-square with Yates' continuity correction or Fisher’s exact test where any observed cell count was fewer than five, as appropriate. All analyses were performed on a complete-case basis without imputation, with available denominators reported for variables with missing data. A two-sided p-value <0.05 was considered statistically significant. Associations with 30-day mortality were assessed using univariable logistic regression and are reported as odds ratios (ORs) with 95% confidence intervals (CIs). A multivariable logistic regression model included age (per decade), ASA grade (I, II, III, IV-V), preoperative sepsis category (none, SIRS/infection, sepsis/septic shock), emergency laparotomy, and generalized peritonitis/gross contamination. Covariates were selected on clinical grounds, prioritizing parsimony and avoiding redundancy among correlated measures of comorbidity, frailty, and physiological derangement, rather than because of limited statistical power; with eight degrees of freedom and 156 observed deaths, the events-per-variable ratio was 19.5. Collinearity was assessed using variance inflation factors and linearity in the logit for age by comparison with a model including a quadratic age term. The global significance of the multi-level ASA and sepsis terms was assessed using likelihood-ratio tests. Model discrimination was evaluated using the area under the receiver operating characteristic curve (AUROC), with optimism correction using Harrell’s bootstrap procedure with 2,000 resamples. Post hoc sensitivity analyses were performed to examine the robustness of the primary model to alternative specifications and to missing data. These analyses were conducted post hoc and are solely reported as exploratory.

Four risk-prediction scores - NELA, ESAS, P-POSSUM, and SRS - were examined in a secondary exploratory analysis. NELA was assessed among eligible emergency-laparotomy patients, whereas ESAS, P-POSSUM, and SRS were assessed across the overall EGS cohort where calculable. Discrimination was evaluated using AUROC with bootstrap-derived 95% CIs (2,000 resamples). Formal assessment of calibration, observed-to-expected mortality, and model recalibration was not performed. 

All analyses were performed using IBM SPSS version 30 (IBM Corp., Armonk, NY, USA).

## Results

Cohort composition

Between January 2020 and July 2025, 2,506 emergency general surgical cases were screened. After application of the predefined eligibility criteria, 466 were excluded, leaving 2,040 patients in the final analysis (Figure 1). Overall, 156 patients (7.6%) died within 30 days of surgery. Of these 2,040 patients, 415 (20.3%) underwent an emergency laparotomy, as defined in the Methods. NELA eligibility could be determined in 394 of these 415 patients (94.9%); of these, 361 (91.6%) met the published NELA case definition, 33 did not, and eligibility could not be confidently established in the remaining 21, who were excluded from the NELA-eligible subgroup rather than assumed eligible. Thirty-day mortality was 12.5% (52/415) among patients undergoing emergency laparotomy and 11.6% (42/361) among NELA-eligible patients.

**Figure 1:**
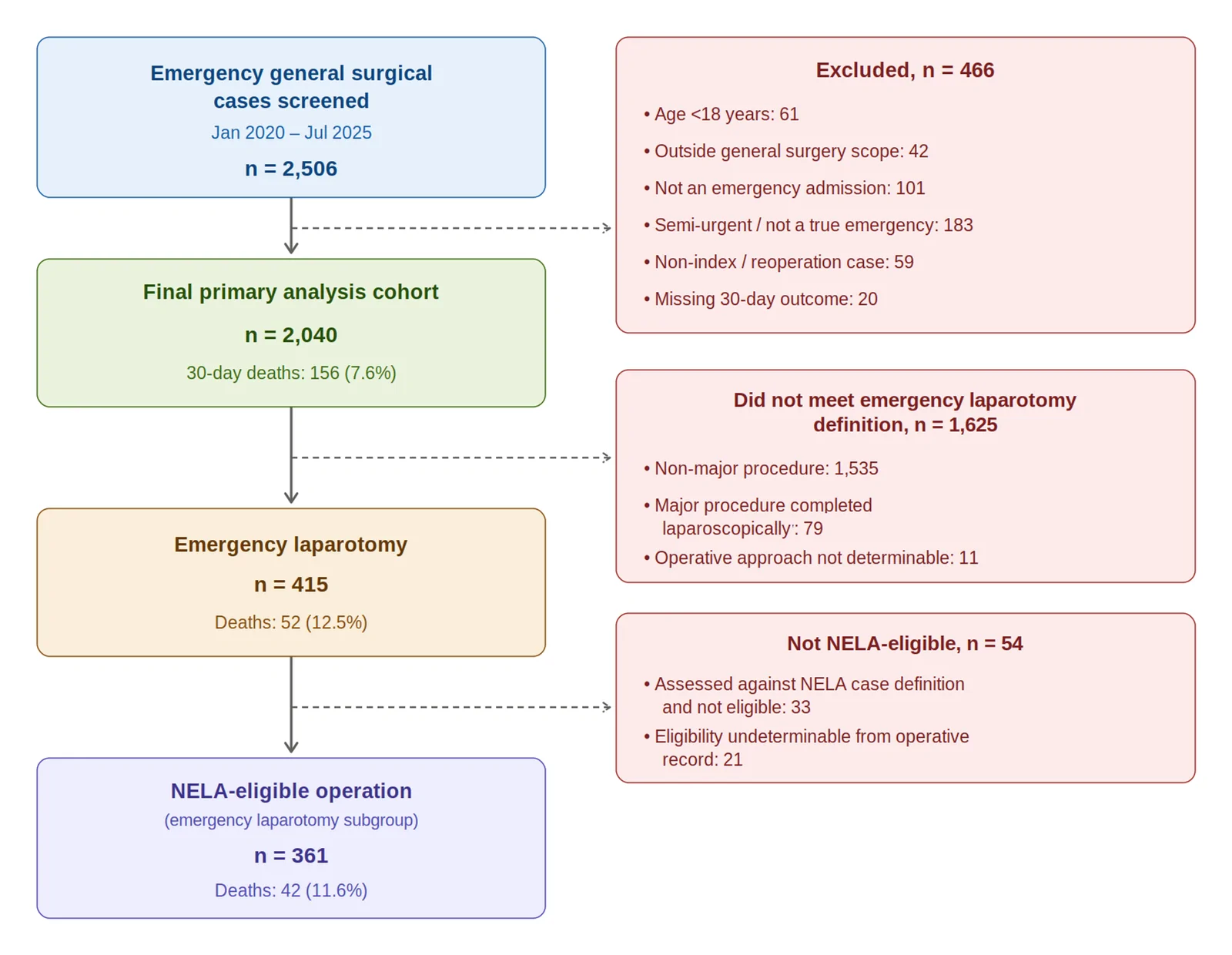
Cohort flow diagram: derivation of the final primary analysis cohort and predefined operative subgroups Image Credits: Alexis Terras, Nikolaos Machairas using Canva (Canva Pty Ltd., Sydney, Australia)

Baseline demographic and clinical characteristics

Baseline demographic, comorbidity, and preoperative physiological characteristics are summarized in Table 1. Non-survivors were significantly older (mean age 64.6 ± 18.4 vs. 51.2 ± 19.6 years, p<0.0001) and had significantly higher ASA grade, Charlson Comorbidity Index, and Clinical Frailty Scale scores (all p<0.0001). Hypertension, diabetes, chronic steroid use, and a more advanced preoperative sepsis category were all significantly more prevalent among non-survivors, as was preoperative vasopressor requirement and need for preoperative mechanical ventilation.

**Table 1:** Baseline demographic and clinical characteristics Continuous variables were compared using Welch's t-test (reported as t) or the Mann–Whitney U test (reported as U), as appropriate for distribution; categorical variables were compared using the chi-squared test with Yates' continuity correction (reported as χ²). Preoperative sepsis category was compared as a four-level variable using the chi-squared test (df=3). Statistical significance was defined as p<0.05 (two-sided) and significant p-values are marked with an asterisk (*). ASA: American Society of Anesthesiologists; BMI: body mass index; IHD: ischemic heart disease; IQR: interquartile range; SD: standard deviation; COPD: chronic obstructive pulmonary disease; SIRS: systemic inflammatory response syndrome

Variable	Overall (n=2,040)	Survivors (n=1,884)	Non-survivors (n=156)	Test statistic	p-value	Test
Age, years — mean ± SD	52.3 ± 19.8	51.2 ± 19.6	64.6 ± 18.4	t = −8.65	<0.0001*	Welch's t-test
Sex, female — n (%)	1063 (52.1%)	979 (52.0%)	84 (53.8%)	χ² = 0.14	0.712	Chi-squared (Yates)
BMI, kg/m2 — mean ± SD	27.1 ± 5.6	27.1 ± 5.6	27.0 ± 5.5	t = 0.06	0.95	Welch's t-test
ASA grade — median (IQR)	2 (2,3)	2 (2,3]	3 (2-40	U = 83,718.5	<0.0001*	Mann–Whitney U
Charlson Comorbidity Index — median (IQR)	1.0 (0.0–2.0)	1.0 (0.0–2.0)	2.0 (1.0–3.00	U = 104,291.5	<0.0001*	Mann–Whitney U
Clinical Frailty Scale — median (IQR)	3.0 (2.0–5.0)	3.0 (2.0–5.0)	5.0 (3.0–6.0)	U = 89,622.0	<0.0001*	Mann–Whitney U
Functional dependence — n (%)	155 (7.6%)	143 (7.6%)	12 (7.7%)	χ² = 0.00	1	Chi-squared (Yates)
COPD — n (%)	149 (7.3%)	138 (7.3%)	11 (7.1%)	χ² = 0.00	1	Chi-squared (Yates)
Chronic kidney disease — n (%)	157 (7.7%)	146 (7.7%)	11 (7.1%)	χ² = 0.03	0.874	Chi-squared (Yates)
IHD / heart failure — n (%)	225 (11.0%)	198 (10.5%)	27 (17.3%)	χ² = 6.11	0.013*	Chi-squared (Yates)
Atrial fibrillation — n (%)	121 (5.9%)	109 (5.8%)	12 (7.7%)	χ² = 0.63	0.428	Chi-squared (Yates)
Diabetes mellitus — n (%)	230 (11.3%)	201 (10.7%)	29 (18.6%)	χ² = 8.26	0.004*	Chi-squared (Yates)
Hypertension — n (%)	462 (22.6%)	406 (21.5%)	56 (35.9%)	χ² = 16.12	<0.0001*	Chi-squared (Yates)
Disseminated cancer — n (%)	118 (5.8%)	104 (5.5%)	14 (9.0%)	χ² = 2.55	0.11	Chi-squared (Yates)
Chronic steroid use — n (%)	70 (3.4%)	55 (2.9%)	15 (9.6%)	χ² = 17.53	<0.0001*	Chi-squared (Yates)
Anticoagulation — n (%)	116 (5.7%)	104 (5.5%)	12 (7.7%)	χ² = 0.89	0.344	Chi-squared (Yates)
Preoperative sepsis category — n (%)				χ² = 175.90 (df=3)	<0.0001*	Chi-squared
None	1141 (55.9%)	1100 (58.4%)	41 (26.3%)	-	-	-
SIRS/infection	617 (30.2%)	561 (29.8%)	56 (35.9%)	-	-	-
Sepsis	214 (10.5%)	186 (9.9%)	28 (17.9%)	-	-	-
Septic shock	68 (3.3%)	37 (2.0%)	31 (19.9%)	-	-	-
Vasopressor requirement — n (%)	40 (2.0%)	22 (1.2%)	18 (11.5%)	χ² = 75.30	<0.0001*	Chi-squared (Yates)
Preop. ventilation (48h) — n (%)	14 (0.7%)	6 (0.3%)	8 (5.1%)	χ² = 42.10	<0.0001*	Chi-squared (Yates)

Preoperative laboratory parameters

Preoperative laboratory parameters are summarized in Table 2. Non-survivors had significantly higher white cell counts, higher C-reactive protein, higher urea and creatinine, lower albumin, lower sodium, higher INR, and higher lactate (all p<0.05); hemoglobin and platelet count did not differ significantly.

**Table 2:** Preoperative laboratory parameters: overall cohort, survivors, and non-survivors Continuous variables were compared using the Mann–Whitney U test (reported as U); dichotomized threshold variables were compared using the chi-squared test with Yates' continuity correction (reported as χ²). Statistical significance was defined as p<0.05 (two-sided) and significant p-values are marked with an asterisk (*). Denominators vary by parameter owing to incomplete data and are given for each variable. CRP, C-reactive protein; INR, international normalized ratio; IQR, interquartile range; WCC, white cell count

Variable	Overall (n=2,040)	Survivors (n=1,884)	Non-survivors (n=156)	Test statistic	p-value	Test
Hemoglobin, g/dL — median (IQR) (n=2034)	12.9 (11.8–14.0)	12.9 (11.8–14.0)	12.8 (11.7–13.8)	U = 155,836.0	0.185	Mann–Whitney U
WCC, x10⁹/L — median (IQR) (n=2031)	10.6 (8.1–13.0)	10.4 (8.0–12.9)	12.1 (9.6–14.5)	U = 113,214.5	<0.0001*	Mann–Whitney U
WCC >15 x10⁹/L — n (%) (n=2031)	244 (12.0%)	214 (11.4%)	30 (19.2%)	χ² = 7.60	0.006*	Chi-squared (Yates)
Platelets, x10⁹/L — median (IQR) (n=2031)	250.0 (205.0–297.0)	250.0 (204.0–297.0)	248.5 (210.2–297.0)	U = 142,215.5	0.741	Mann–Whitney U
CRP, mg/L — median (IQR) (n=1996)	83.0 (37.0–129.0)	81.0 (35.0–125.0)	105.0 (61.5–178.5)	U = 104,896.5	<0.0001*	Mann–Whitney U
Urea, mg/dL — median (IQR) (n=2031)	46.0 (32.0–62.0)	46.0 (31.0–61.0)	55.0 (36.0–69.0)	U = 119,476.5	<0.001*	Mann–Whitney U
Creatinine, mg/dL — median (IQR) (n=2028)	1.1 (0.8–1.4)	1.1 (0.8–1.4)	1.2 (0.9–1.5)	U = 122,386.0	<0.001*	Mann–Whitney U
Albumin, g/L — median (IQR) (n=1715)	36.0 (32.0–39.0)	36.0 (32.0–39.0)	34.0 (30.0–38.0)	U = 121,498.0	<0.001*	Mann–Whitney U
Sodium, mmol/L — median (IQR) (n=2025)	136.0 (134.0–139.0)	136.0 (134.0–139.0)	135.0 (133.0–138.0)	U = 160,311.0	0.019*	Mann–Whitney U
INR — median (IQR) (n=1848)	1.1 (1.0–1.2)	1.1 (1.0–1.2)	1.1 (1.0–1.3)	U = 92,828.0	<0.0001*	Mann–Whitney U
Lactate, mmol/L — median (IQR) (n=1,456)	2.0 (1.2–2.7)	1.9 (1.2–2.6)	2.5 (1.9–3.3)	U = 52,964.0	<0.0001*	Mann–Whitney U
Lactate >2 mmol/L — n (%) (n=1,456)	683 (46.9%)	599 (44.9%)	84 (68.9%)	χ² = 24.79	<0.0001*	Chi-squared (Yates)

Disease and procedure profile

The distribution of admission diagnoses, surgical approach, procedure type, and intraoperative findings is presented in Table 3. Appendicitis and perianal/perirectal sepsis were significantly less common among non-survivors, reflecting their comparatively benign course, whereas perforation/peritonitis, bowel ischemia, and soft-tissue/necrotizing infection were all significantly more common among non-survivors. Emergency laparotomy, bowel resection, stoma formation, generalized peritonitis or gross intraoperative contamination, fecal contamination, and intraoperatively identified bowel ischemia or necrosis were all significantly more frequent among non-survivors (all p<0.05). An open surgical approach was significantly more common among non-survivors (62.2% (97/156) vs. 47.0% (885/1884), p<0.001), while a laparoscopic approach was significantly less common (20.5% (32/156) vs. 38.3% (722/1884), p<0.0001)); conversion from laparoscopic to open surgery and a local/minor procedure did not differ significantly between groups.

**Table 3:** Admission diagnosis, surgical approach, procedure performed, and intraoperative findings Each category was compared between survivors and non-survivors using the chi-squared test with Yates' continuity correction (reported as χ²), except where any observed cell count was fewer than five, in which case Fisher's exact test was used; Fisher's exact test yields no test statistic and these rows are marked accordingly. Statistical significance was defined as p<0.05 (two-sided) and significant p-values are marked with an asterisk (*).

Variable	Overall (n=2,040)	Survivors (n=1,884)	Non-survivors (n=156)	Test statistic	p-value	Test
Primary Diagnosis — n (%)						
Appendicitis	430 (21.1%)	427 (22.7%)	3 (1.9%)	χ² = 36.02	<0.0001*	Chi-squared (Yates)
Cholecystitis/biliary	282 (13.8%)	275 (14.6%)	7 (4.5%)	χ² = 11.53	<0.001*	Chi-squared (Yates)
Bowel obstruction	270 (13.2%)	253 (13.4%)	17 (10.9%)	χ² = 0.60	0.439	Chi-squared (Yates)
Hernia	184 (9.0%)	175 (9.3%)	9 (5.8%)	χ² = 1.77	0.184	Chi-squared (Yates)
Perforation/peritonitis	150 (7.4%)	127 (6.7%)	23 (14.7%)	χ² = 12.39	<0.001*	Chi-squared (Yates)
Diverticulitis	149 (7.3%)	139 (7.4%)	10 (6.4%)	χ² = 0.08	0.775	Chi-squared (Yates)
Bowel ischemia	57 (2.8%)	28 (1.5%)	29 (18.6%)	χ² = 148.94	<0.0001*	Chi-squared (Yates)
Soft tissue infection/abscess	213 (10.4%)	180 (9.6%)	33 (21.2%)	χ² = 19.51	<0.0001*	Chi-squared (Yates)
Perianal/perirectal	130 (6.4%)	128 (6.8%)	2 (1.3%)	— (exact test)	0.003*	Fisher's exact
GI bleeding/ulcer	98 (4.8%)	86 (4.6%)	12 (7.7%)	χ² = 2.44	0.119	Chi-squared (Yates)
Cancer-related emergency	51 (2.5%)	44 (2.3%)	7 (4.5%)	χ² = 1.92	0.165	Chi-squared (Yates)
Other	26 (1.3%)	22 (1.2%)	4 (2.6%)	— (exact test)	0.132	Fisher's exact
Surgical approach — n (%)						
Open	982 (48.1%)	885 (47.0%)	97 (62.2%)	χ² = 12.74	<0.001*	Chi-squared (Yates)
Laparoscopic	754 (37.0%)	722 (38.3%)	32 (20.5%)	χ² = 18.86	<0.0001*	Chi-squared (Yates)
Laparoscopic converted to open	120 (5.9%)	108 (5.7%)	12 (7.7%)	χ² = 0.68	0.411	Chi-squared (Yates)
Local/minor procedure/other	184 (9.0%)	169 (9.0%)	15 (9.6%)	χ² = 0.02	0.901	Chi-squared (Yates)
High-risk operative features — n (%)						
Emergency laparotomy	415 (20.3%)	363 (19.3%)	52 (33.3%)	χ² = 16.73	<0.0001*	Chi-squared (Yates)
Bowel resection performed	290 (14.2%)	246 (13.1%)	44 (28.2%)	χ² = 25.88	<0.0001*	Chi-squared (Yates)
Stoma formation (any)	217 (10.6%)	183 (9.7%)	34 (21.8%)	χ² = 20.87	<0.0001*	Chi-squared (Yates)
Generalized peritonitis/gross contamination	367 (18.0%)	311 (16.5%)	56 (35.9%)	χ² = 35.41	<0.0001*	Chi-squared (Yates)
Fecal contamination	129 (6.3%)	110 (5.8%)	19 (12.2%)	χ² = 8.74	0.003*	Chi-squared (Yates)
Bowel ischemia / necrosis (intraoperative)	145 (7.1%)	109 (5.8%)	36 (23.1%)	χ² = 62.65	<0.0001*	Chi-squared (Yates)

Postoperative course and outcomes

Postoperative outcomes are summarized in Table 4. In-hospital mortality was 6.7% (136/2,040), accounting for 87.2% (136/156) of all 30-day deaths; 20 deaths occurred after discharge but within 30 days of surgery. Any postoperative complication occurred in 534 patients (26.2%) and a major complication (Clavien-Dindo grade ≥III) in 259 (12.7%). Because death constitutes Clavien-Dindo grade V, complication rates were not compared inferentially between survivors and non-survivors.

**Table 4:** Postoperative outcomes: overall cohort, survivors, and non-survivors ᵃReadmission is assessed among the 1,904 patients discharged alive (30-day survivors, n=1,884, plus the 20 patients who died after discharge within 30 days); readmission was impossible for the 136 patients who died in-hospital. Categorical variables were compared using the chi-squared test with Yates' continuity correction (reported as χ²); length-of-stay variables were compared using the Mann–Whitney U test (reported as U). Statistical significance was defined as p<0.05 (two-sided) and significant p-values are marked with an asterisk (*). In-hospital mortality and complication rates were not compared inferentially between survivors and non-survivors because death constitutes Clavien–Dindo grade V. Total hospital length of stay is likewise reported descriptively and was not compared inferentially, as non-survivors have by definition a shorter period during which length of stay accrues (competing-risk bias). Rows not compared inferentially are shown as "—". ICU: intensive care unit

Variable	Overall (n=2,040)	Survivors (n=1,884)	Non-survivors (n=156)	Test statistic	p-value	Test
In-hospital mortality	136 (6.7%)	-	136 (87.2%)	—	—	Not tested (see legend)
Any postoperative complication	534 (26.2%)	378 (20.1%)	156 (100%, by definition)	—	—	Not tested (see legend)
Major complication, Clavien-Dindo ≥III	259 (12.7%)	103 (5.5%)	156 (100%, by definition)	—	—	Not tested (see legend)
Postoperative sepsis	342 (16.8%)	257 (13.6%)	85 (54.5%)	χ² = 169.34	<0.0001*	Chi-squared (Yates)
ICU admission	564 (27.6%)	439 (23.3%)	125 (80.1%)	χ² = 229.75	<0.0001*	Chi-squared (Yates)
ICU length of stay, days	3.0 (2.0–5.0)	3.0 (2.0–4.0)	4.0 (2.0–10.0)	U = 22,187.5	<0.001*	Mann–Whitney U
Reoperation within 30 days	75 (3.7%)	53 (2.8%)	22 (14.1%)	χ² = 48.71	<0.0001*	Chi-squared (Yates)
Radiological intervention	105 (5.1%)	50 (2.7%)	55 (35.3%)	χ² = 307.02	<0.0001*	Chi-squared (Yates)
Readmission within 30 daysᵃ	133/1,904 (7.0%)	128/1,884 (6.8%)	5/20 (25.0%)	χ² = 7.49	0.006*	Chi-squared (Yates)
Total hospital length of stay, days	6.0 (5.0–8.0)	6.0 (5.0–8.0)	6.0 (4.0–14.2)	—	—	Not tested (see legend)

Postoperative sepsis, ICU admission, and reoperation within 30 days were more frequent among non-survivors (all p<0.0001). Reoperation was required in 75 patients (3.7%), of whom 22 (29.3%) died within 30 days. ICU length of stay was longer among non-survivors (4.0 (2.0-10.0) vs. 3.0 (2.0-4.0) days, p<0.001), whereas total hospital length of stay is reported descriptively and was not compared inferentially between groups.

Mortality by predefined subgroup

Diagnosis-specific mortality ranged from 0.7% (3/430) for appendicitis and 1.5% (2/130) for perianal/perirectal sepsis to 50.9% (29/57) for bowel ischemia (Figure 2). Mortality also increased in a graded fashion across ASA grade, from 2.0% (8/403) for ASA I to 52.9% (9/17) for ASA V, and across preoperative sepsis category, from 3.6% (41/1141) in patients without sepsis to 45.6% (31/68) in those presenting with septic shock (Figure 3).

**Figure 2:**
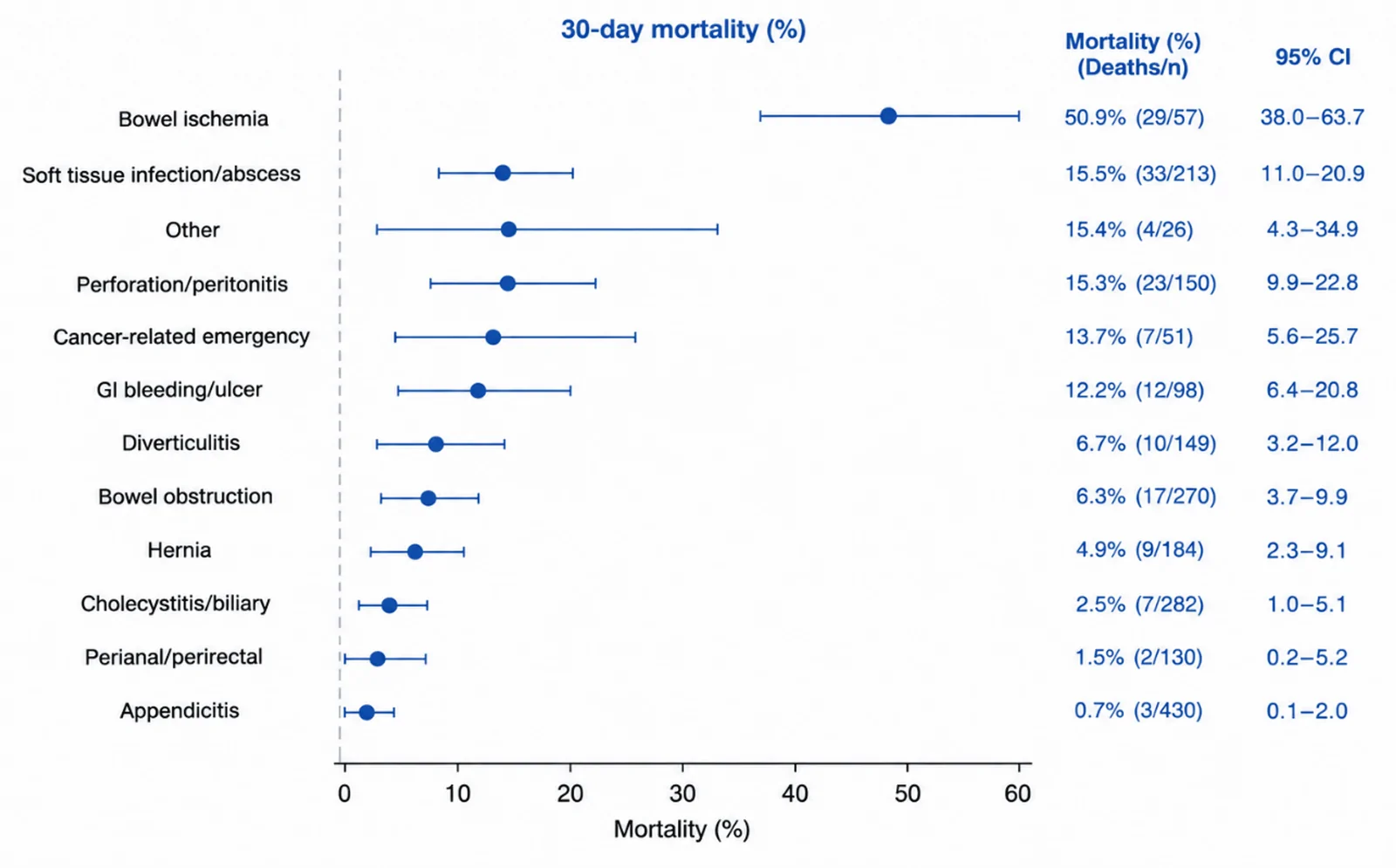
Thirty-day mortality by primary diagnosis group

**Figure 3:**
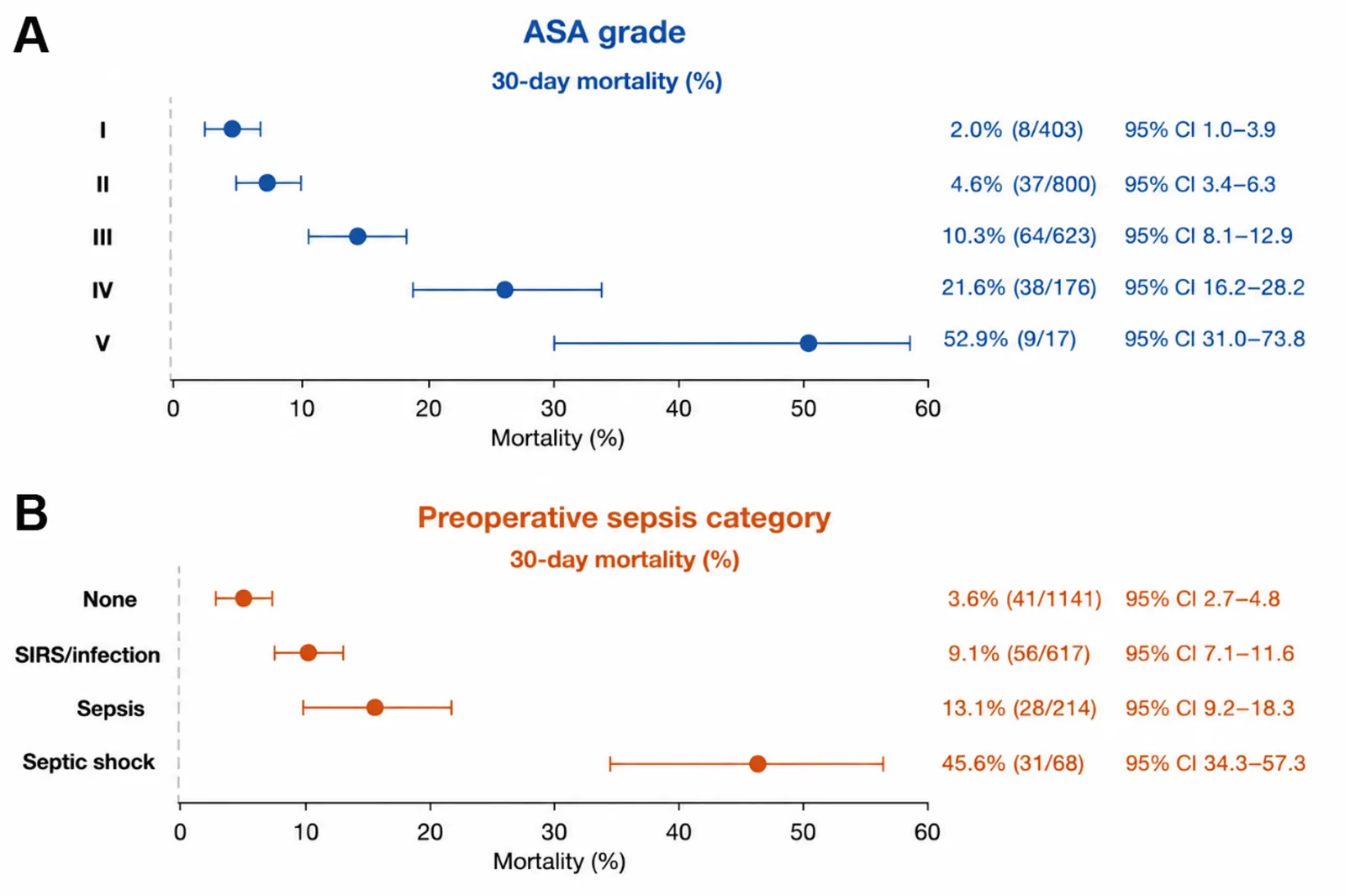
Thirty-day mortality by ASA grade and preoperative sepsis category A: 30-day mortality by ASA grade; B: 30-day mortality by preoperative sepsis category ASA: American Society of Anesthesiologists

Predictors of 30-day mortality

Univariable logistic regression results for all candidate variables are presented in Table 5. Older age, higher ASA grade, greater comorbidity burden, frailty, hypertension, diabetes mellitus, ischemic heart disease/heart failure, chronic steroid use, increasing preoperative sepsis severity, emergency laparotomy, NELA eligibility, bowel resection, stoma formation, generalized peritonitis, intraoperative bowel ischemia or necrosis, and derangement in each of the recorded preoperative laboratory parameters (higher white cell count, C-reactive protein, urea, creatinine, and international normalized ratio; higher lactate; and lower albumin and sodium) were all associated with higher 30-day mortality (all p<0.05).

**Table 5:** Univariable predictors of 30-day mortality Odds ratios were derived from separate univariable binary logistic regression models, one per variable. p-values and test statistics are from the Wald test, reported as the Wald χ². Statistical significance was defined as p<0.05 (two-sided), and significant p-values are marked with an asterisk (*); all associations shown reached statistical significance. CI: confidence interval; OR: odds ratio; ASA: American Society of Anesthesiologists; BMI: body mass index; IHD: ischemic heart disease; SIRS: systemic inflammatory response syndrome; NELA: National Emergency Laparotomy Audit

Variable / term	OR	95% CI	Test statistic	p	Test
Demographic and baseline risk					
Age, per 10 years	1.42	1.30–1.55	Wald χ² = 60.73	<0.0001*	Wald test, logistic regression
ASA II vs I	2.39	1.10–5.19	Wald χ² = 4.89	0.027*	Wald test, logistic regression
ASA III vs I	5.65	2.68–11.92	Wald χ² = 20.70	<0.0001*	Wald test, logistic regression
ASA IV vs I	13.6	6.19–29.86	Wald χ² = 42.28	<0.0001*	Wald test, logistic regression
ASA V vs I	55.55	17.04–181.12	Wald χ² = 44.38	<0.0001*	Wald test, logistic regression
Charlson Comorbidity Index, per point	1.44	1.29–1.60	Wald χ² = 44.96	<0.0001*	Wald test, logistic regression
Clinical Frailty Scale, per point	1.51	1.38–1.66	Wald χ² = 75.93	<0.0001*	Wald test, logistic regression
Hypertension (yes vs no)	2.04	1.44-2.88	Wald χ² = 16.37	<0.0001*	Wald test, logistic regression
Diabetes mellitus (yes vs no)	1.91	1.24-2.94	Wald χ² = 8.77	0.003*	Wald test, logistic regression
Chronic steroid use (yes vs no)	3.54	1.95–6.42	Wald χ² = 17.26	<0.0001*	Wald test, logistic regression
IHD/heart failure (yes vs no)	1.78	1.15-2.77	Wald χ² = 6.62	0.010*	Wald test, logistic regression
Preoperative physiology					
SIRS/infection vs None	2.68	1.77–4.06	Wald χ² = 21.60	<0.0001*	Wald test, logistic regression
Sepsis vs None	4.04	2.44–6.69	Wald χ² = 29.35	<0.0001*	Wald test, logistic regression
Septic shock vs None	22.48	12.71–39.75	Wald χ² = 114.54	<0.0001*	Wald test, logistic regression
WCC, per unit	1.11	1.06–1.16	Wald χ² = 21.30	<0.0001*	Wald test, logistic regression
CRP, per 10 mg/L	1.08	1.06–1.11	Wald χ² = 40.70	<0.0001*	Wald test, logistic regression
Urea, per 10 mg/dL (n=2,031)	1.17	1.08–1.26	Wald χ² = 15.85	<0.0001*	Wald test, logistic regression
Creatinine, per 1 mg/dL (n=2,028)	1.98	1.37–2.88	Wald χ² = 13.02	<0.001*	Wald test, logistic regression
Albumin, per 5 g/L (n=1,715)	0.75	0.63–0.88	Wald χ² = 12.04	<0.001*	Wald test, logistic regression
Sodium, per 5 mmol/L (n=2,025)	0.73	0.58–0.93	Wald χ² = 6.66	0.010*	Wald test, logistic regression
INR, per 0.1 unit (n=1,848)	1.31	1.18–1.47	Wald χ² = 24.25	<0.0001*	Wald test, logistic regression
Lactate, per 1 mmol/L (n=1,456)	1.81	1.51–2.16	Wald χ² = 42.08	<0.0001*	Wald test, logistic regression
Operative factors					
Emergency laparotomy (yes vs no)	2.1	1.47–2.98	Wald χ² = 16.96	<0.0001*	Wald test, logistic regression
NELA-eligible operation (yes vs no)	1.89	1.30–2.75	Wald χ² = 10.99	<0.001*	Wald test, logistic regression
Bowel resection performed (yes vs no)	2.62	1.80–3.80	Wald χ² = 25.45	<0.0001*	Wald test, logistic regression
Stoma formation (yes vs no)	2.59	1.72–3.90	Wald χ² = 20.75	<0.0001*	Wald test, logistic regression
Generalized peritonitis (yes vs no)	2.83	2.00–4.02	Wald χ² = 34.19	<0.0001*	Wald test, logistic regression
Bowel ischemia/necrosis, intraop (yes vs no)	4.89	3.21–7.43	Wald χ² = 54.88	<0.0001*	Wald test, logistic regression

On multivariable analysis adjusting simultaneously for age, ASA grade, preoperative sepsis category, emergency laparotomy, and generalized peritonitis, ASA grade and preoperative sepsis category remained independently associated with 30-day mortality (Table 6). ASA grade was not recorded in 21 patients (1.0%), all of whom survived; the multivariable model was therefore fitted on 2,019 patients. In sensitivity analyses assigning these patients to the modal ASA category, and to ASA IV-V as a worst case, adjusted estimates were materially unchanged (ASA IV-V aOR 4.63 and 3.72, respectively, versus 4.53 in the complete-case analysis), indicating that the complete-case exclusion did not bias the adjusted estimates.

**Table 6:** Multivariable predictors of 30-day mortality (adjusted for age, ASA grade, preoperative sepsis category, emergency laparotomy, and generalized peritonitis; n=2,019) ASA grade was not recorded in 21 patients, all survivors. Adjusted odds ratios were derived from a single multivariable binary logistic regression model. p-values and test statistics are from the Wald test, reported as the Wald χ². Statistical significance was defined as p<0.05 (two-sided) and significant p-values are marked with an asterisk (*). aOR: adjusted odds ratio; ASA: American Society of Anesthesiologists; CI: confidence interval; SIRS: systemic inflammatory response syndrome

Variable / term	Adjusted OR	95% CI	Test statistic	p	Test
ASA II vs I	1.65	0.74–3.70	Wald χ² = 1.48	0.224	Wald test, logistic regression
ASA III vs I	2.53	1.05–6.08	Wald χ² = 4.27	0.039*	Wald test, logistic regression
ASA IV–V vs I	4.53	1.67–12.30	Wald χ² = 8.81	0.003*	Wald test, logistic regression
Sepsis: SIRS/infection vs None	1.95	1.26–3.03	Wald χ² = 8.85	0.003*	Wald test, logistic regression
Sepsis: Sepsis/septic shock vs None	3.52	2.10–5.88	Wald χ² = 22.96	<0.0001*	Wald test, logistic regression
Emergency laparotomy	0.7	0.45–1.07	Wald χ² = 2.75	0.098	Wald test, logistic regression
Generalized peritonitis	1.49	0.97–2.30	Wald χ² = 3.34	0.068	Wald test, logistic regression
Age, per 10 years	1.13	1.00–1.28	Wald χ² = 3.56	0.059	Wald test, logistic regression

Compared with ASA I, the adjusted odds of death were higher for ASA III (aOR 2.53, 95% CI 1.05-6.08) and ASA IV-V (aOR 4.53, 95% CI 1.67-12.30). Compared with patients without preoperative sepsis, adjusted odds of death were higher for SIRS/infection (aOR 1.95, 95% CI 1.26-3.03) and sepsis/septic shock (aOR 3.52, 95% CI 2.10-5.88). Age, emergency laparotomy, and generalized peritonitis did not reach statistical significance threshold after adjustment. Emergency laparotomy additionally reversed direction between the univariable and adjusted estimates (unadjusted OR 2.10; aOR 0.70, 95% CI 0.45-1.07, p=0.098). This reversal is consistent with confounding by indication: patients selected for laparotomy are substantially sicker, and once ASA grade and preoperative sepsis category are held constant, the residual association with laparotomy itself is attenuated, with the point estimate moving below unity. Because the confidence interval crosses 1, these data neither demonstrate nor exclude a protective effect of laparotomy within severity strata, and the adjusted estimate should not be read as evidence that laparotomy reduces mortality. The remaining non-significant associations may similarly reflect correlation between covariates, differences in case-mix, limited precision, or the selected model specification.

Sensitivity analyses

Variance inflation factors were below 3.3 for all covariates in the primary model (highest, ASA III: 3.24), indicating no substantial collinearity. Several post hoc specifications were examined. Adding bowel ischemia as the primary diagnosis produced an independent association with mortality (aOR 4.71, 95% CI 2.29-9.69, p<0.001) and attenuated the estimates for ASA IV-V (aOR 3.04, 95% CI 1.08-8.58) and sepsis or septic shock (aOR 2.63, 95% CI 1.50-4.60), with improved model fit (AIC 970.4 vs 986.6). Replacing ASA grade with the Charlson Comorbidity Index and Clinical Frailty Scale rendered generalized peritonitis statistically significant (aOR 1.54, 95% CI 1.01-2.36, p=0.047), while age remained non-significant. Adding creatinine (aOR 0.95, 95% CI 0.62-1.44) or albumin (aOR 0.95 per 5 g/L, 95% CI 0.79-1.13) did not produce independent associations or improve fit. Data completeness, case mix, and mortality were comparable between the retrospective and prospective phases (30-day mortality 7.3% vs 8.1%, p=0.554). A pandemic-period term (January 2020-April 2022) was not independently associated with mortality (aOR 0.74, 95% CI 0.51-1.07, p=0.112) and left the other adjusted estimates essentially unchanged.

Risk-prediction scores

Risk scores were calculated on a complete-case basis, where all variables required for each instrument were available; no imputation was performed. Discrimination was moderate across the four scores evaluated, with AUROCs ranging from 0.66 to 0.72 (Figure 4). ESAS, SRS, and P-POSSUM were calculable in 1,700, 2,019, and 1,991 patients, respectively. The NELA score was calculable in 355 of the 361 confirmed NELA-eligible patients (98.3%); six patients were excluded because one or more required NELA input variables were missing. The 33 emergency laparotomies that were determinable but did not meet the NELA case definition, and the 21 for whom eligibility could not be established, were both excluded from the NELA analysis rather than assumed eligible. Calibration, observed-to-expected mortality ratios, and agreement between predicted and observed risk were not assessed. These exploratory discrimination estimates therefore require confirmation in a prespecified validation study incorporating formal assessment of both discrimination and calibration before the scores are used for absolute risk prediction or benchmarking in Greek EGS practice.

**Figure 4:**
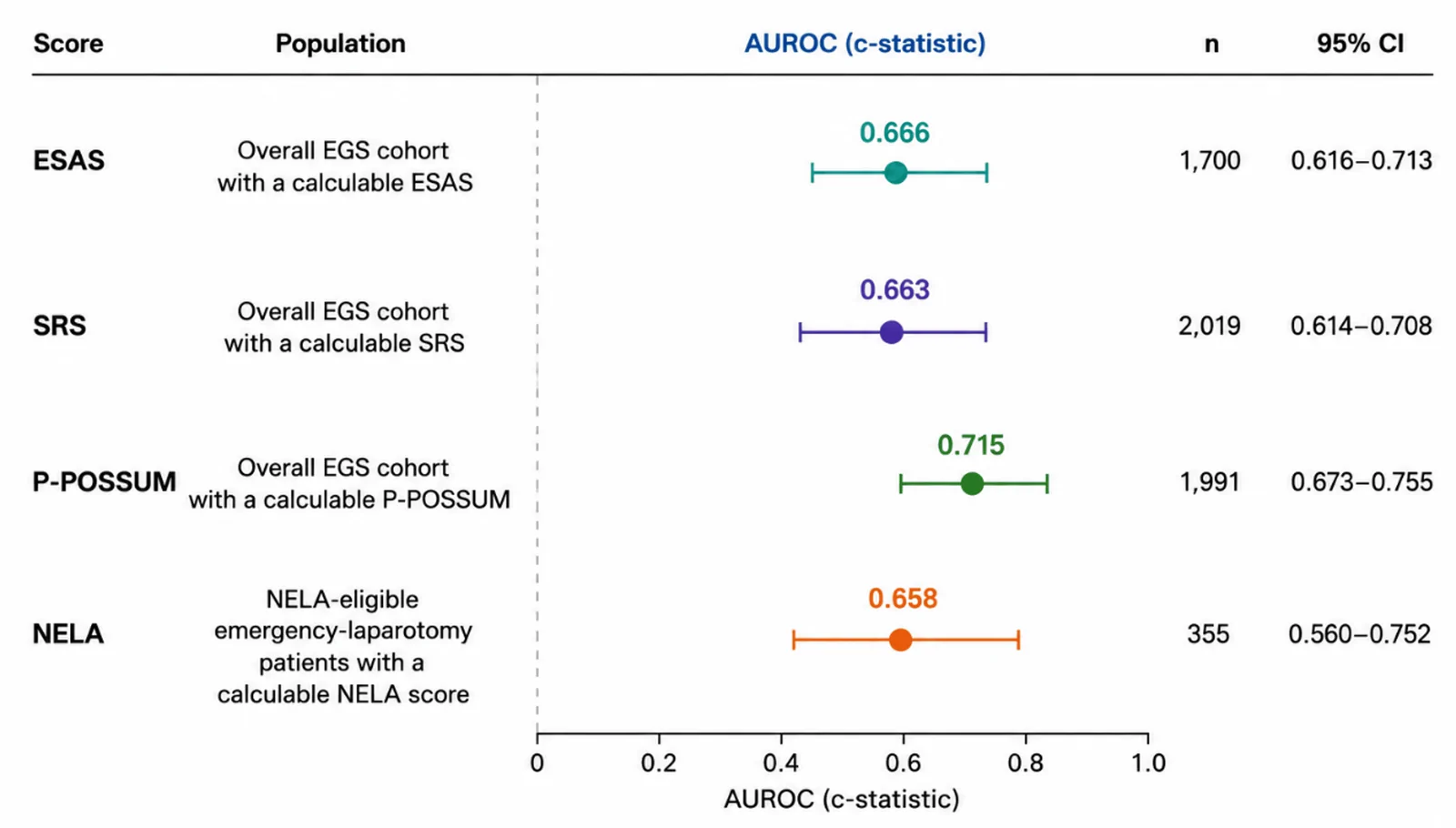
Discrimination (AUROC) of established emergency-surgery risk-prediction scores, overall cohort and emergency laparotomy subgroup AUROC: area under the receiver operating characteristic; CI: confidence interval; ESAS: Emergency Surgery Acuity Score; SRS: Surgical Risk Scale; P-POSSUM: Portsmouth Physiological and Operative Severity Score for the enUmeration of Mortality and morbidity; NELA: National Emergency Laparotomy Audit

## Discussion

In this single-center cohort of 2,040 patients undergoing emergency general surgery at a Greek tertiary academic hospital, 30-day postoperative mortality was 7.6%, increasing to 12.5% among patients undergoing emergency laparotomy and 11.6% among NELA-eligible patients. These rates are consistent with the range reported internationally for EGS and emergency laparotomy populations [4,5,7-9,23-25]. Emergency laparotomy mortality was lower than the 16.3% reported by the multicenter HELAS study [17], although direct comparison is limited by differences in case-mix, study setting, and period. Importantly, whereas contemporary Greek evidence has focused predominantly on emergency laparotomy, the present study extends outcome characterization to the broader spectrum of operatively managed EGS.

Mortality varied markedly and predictably across the cohort: from 0.7% (3/430) for appendicitis to 50.9% (29/57) for bowel ischemia, and along clear, graded relationships with both ASA grade and preoperative sepsis category. Bowel ischemia was the most lethal presentation in this cohort - more than sixfold the cohort average and an order of magnitude above that of appendicitis. This is consistent with published experience: contemporary series report 30-day mortality after acute mesenteric ischemia as high as 50% in the acute setting, with overall mortality conventionally cited at 50-70%, and single-center surgical series reporting figures between 21.4% and 55.9% depending on etiology and case selection [26,27]. Our figure therefore reflects the intrinsic lethality of the condition rather than a local outlier. On multivariable analysis, ASA grade and preoperative sepsis category were the variables that remained independently associated with 30-day mortality in the model, while age, emergency laparotomy, and generalized peritonitis - all significant on univariable analysis - did not survive adjustment. This pattern is broadly consistent with HELAS's identification of age, ASA grade, functional status, and sepsis severity as predictors of death after emergency laparotomy in a larger, multicenter Greek population [17]. This finding also echoes, while partly refining, prior international literature on risk factors for adverse outcome after emergency general surgery. In a large Danish cohort of 5,255 unselected gastrointestinal operations, Sørensen et al. identified patient-, disease-, treatment-, and organization-related predictors of mortality and complications spanning a similarly broad set of domains [28]. Akinbami et al., in a single-center US cohort of 819 emergency general surgery patients with a comparable 30-day mortality of 8.9%, found age, sex, hyperglycemia, renal impairment, hypoalbuminemia, operative duration, and smoking to be independent predictors of morbidity - a broader set of physiological and procedural variables than those that remained independently significant here [29]. Matsuyama et al. similarly reported diabetes, anemia, and ischemic heart disease as independent risk factors for adverse outcome after emergency surgery in a Japanese cohort [30]. The large US administrative-database analysis by Shah et al. identified age, comorbidity burden, and the specific EGS diagnosis category as predictors of in-hospital mortality and complications at a national level [31], and Spence et al., in a South African multicenter cohort, similarly implicated patient age and postoperative critical care needs among the predictors of adverse events after emergency laparotomy [32]. Frailty specifically has also been shown to be independently associated with mortality and resource use after emergency general surgery in a large population-based Canadian cohort [9]. Clinical Frailty Scale was associated with mortality on univariable analysis in the present cohort but was not included in the multivariable model, given its expected correlation with ASA grade and the Charlson Comorbidity Index; in sensitivity analyses replacing ASA grade with the Charlson Comorbidity Index and Clinical Frailty Scale, frailty retained an independent association with mortality.

A secondary finding was the strong univariable association between chronic steroid use and 30-day mortality (odds ratio 3.5), consistent with a large ACS-NSQIP analysis of over 5 million surgical patients across specialties, in which chronic steroid use remained an independent predictor of both 30-day mortality and major complications after adjustment for age, comorbidity, and procedure type [33]. This association may in part reflect confounding by the underlying disease processes necessitating chronic steroid therapy (e.g., autoimmune or inflammatory disease, transplantation, or malignancy) rather than a direct causal effect of steroid exposure, and was not adjusted for other covariates in this univariable analysis; clarifying its independent contribution to risk would require dedicated multivariable investigation.

As expected, postoperative sepsis, ICU admission, and reoperation were all strongly associated with death. These variables should be interpreted primarily as markers of postoperative clinical trajectory rather than as independent preoperative predictors available at the time of the decision to operate. In-hospital mortality was 6.7% (136/2040) and accounted for 87.2% (136/156) of all 30-day deaths, indicating that a meaningful minority of deaths occurred after discharge but within the 30-day postoperative window. Readmission and length-of-stay outcomes require cautious interpretation in this context, because death competes with both subsequent readmission and prolonged postoperative observation. Direct comparison of these outcomes between survivors and non-survivors is therefore subject to competing-risk bias: non-survivors have, by definition, a shorter period during which such events can be observed. Conditioning on 30-day survival status in this way is conceptually related to the immortal-time problem described in pharmacoepidemiology [34], although the mechanism here is competing risk rather than misclassified person-time. In the present cohort, readmission was impossible for the 87.2% (136/156) of non-survivors who died in-hospital, and among the 20 patients who died after discharge, 25.0% (5/20) were readmitted before death. Similarly, length-of-stay patterns likely reflect a mixture of early deaths during the index admission and more prolonged complicated critical-care courses ending in death. This heterogeneity in timing of death has also been described in emergency abdominal surgery: in a Danish cohort of 754 patients, 72% of 30-day deaths occurred within the first 14 days after surgery, while the remainder occurred later after a more protracted clinical course [35].

Risk-prediction scores were examined descriptively in this analysis. All four showed moderate discrimination, suggesting that they retain some ability to rank patients according to relative risk; calibration was not assessed in the present study and cannot be commented on from these data. A formal, larger multicenter external validation of NELA, P-POSSUM, ACS-NSQIP, and POTTER in Greek emergency laparotomy patients reported substantially better discrimination for these same tools (concordance statistics 0.79-0.85) than the AUROCs observed here, and additionally, found poor calibration for all but a surgeon-adjusted model [18]. The lower discrimination observed in our cohort may reflect genuine differences in case-mix rather than inferior intrinsic score performance. These four instruments differ in their original development populations: NELA was derived and validated specifically in emergency laparotomy patients, closely matching the population in which the Greek multicenter validation above was performed; ESAS was developed in a broader emergency general surgery population; and SRS and P-POSSUM were developed as general operative risk models rather than being specific to emergency or general surgery. Applying ESAS, SRS, and P-POSSUM here across the operatively managed spectrum of EGS, including many lower-acuity procedures (e.g., appendicectomy, cholecystectomy, and minor soft-tissue or perianal procedures), represents a broader and more heterogeneous case-mix than some of these tools were designed for, which may plausibly alter discrimination; the relatively small number of events in our cohort (156 deaths) chiefly affects the precision of these AUROC estimates rather than the point estimates themselves. These findings should be interpreted as preliminary because this study was not designed as a formal score-validation analysis; calibration, observed-to-expected mortality ratios, and local recalibration were beyond the scope of the present analysis.

This study has several inherent limitations. First, its single-center, ambispective design limits generalizability and makes the findings dependent on local case-mix, referral patterns, and clinical practice, while the retrospective component may have introduced information bias; data completeness, case-mix, and mortality were nonetheless comparable between phases (30-day mortality 7.3% vs 8.1%, p=0.554). Second, several laboratory variables had incomplete data, most notably lactate (584/2040, 28.6%), albumin (325/2040, 15.9%), and INR (192/2040, 9.4%), as these parameters are obtained selectively in emergency practice rather than as routine. Missingness was not associated with mortality for albumin (p=0.708) or INR (p=0.533); for lactate, measurement was somewhat more frequent among patients who died (8.4% vs 5.8%, p=0.061), which would attenuate rather than inflate the observed association. Formal SOFA scoring was not undertaken because several components were not consistently documented, and the sepsis categories were assigned from contemporaneous clinical documentation rather than by retrospective application of all Sepsis-3 criteria; they should be interpreted as a pragmatic clinical severity gradient. Third, the multivariable model was deliberately restricted to five constructs for parsimony rather than because the number of events constrained model complexity, and the finding that only ASA grade and sepsis category retained independent associations is conditional on this specification. As shown in the sensitivity analyses, alternative specifications yield different sets of independently significant covariates: variables excluded from the model should therefore not be assumed to lack independent prognostic value. Although collinearity was not substantial, ASA grade encompasses both chronic reserve and acute physiological derangement, and partial overadjustment cannot be excluded. Residual confounding remains possible. Operative urgency and decision-to-operation intervals were derived from the emergency theatre booking record, but the organizational determinants of operative timing - theatre availability, out-of-hours staffing, and competing emergency workload - were not captured. Fourth, the risk-prediction score analysis was exploratory and limited to discrimination; calibration and recalibration were not assessed. Fifth, because the NELA-eligible subgroup was derived from open and converted emergency laparotomies only, it excludes the laparoscopically completed procedures captured by the full NELA case definition, and comparison with NELA and HELAS outcomes is therefore not strictly like-for-like. Finally, the study period encompassed the COVID-19 pandemic and a pandemic-period term added to the multivariable model was not independently associated with mortality (aOR 0.74, 95% CI 0.51-1.07) and left other estimates essentially unchanged.

Future work should extend these observations through prospective multicenter studies encompassing the broader spectrum of EGS practice in Greece. Such studies should enable formal external validation and calibration of existing risk-prediction tools, determine whether local recalibration is required, and establish robust risk-adjusted national benchmarks. Ultimately, validated risk stratification could support identification of high-risk patients, inform perioperative resource allocation, and provide a framework for targeted quality-improvement initiatives in Greek EGS practice (Figure 5).

**Figure 5:**
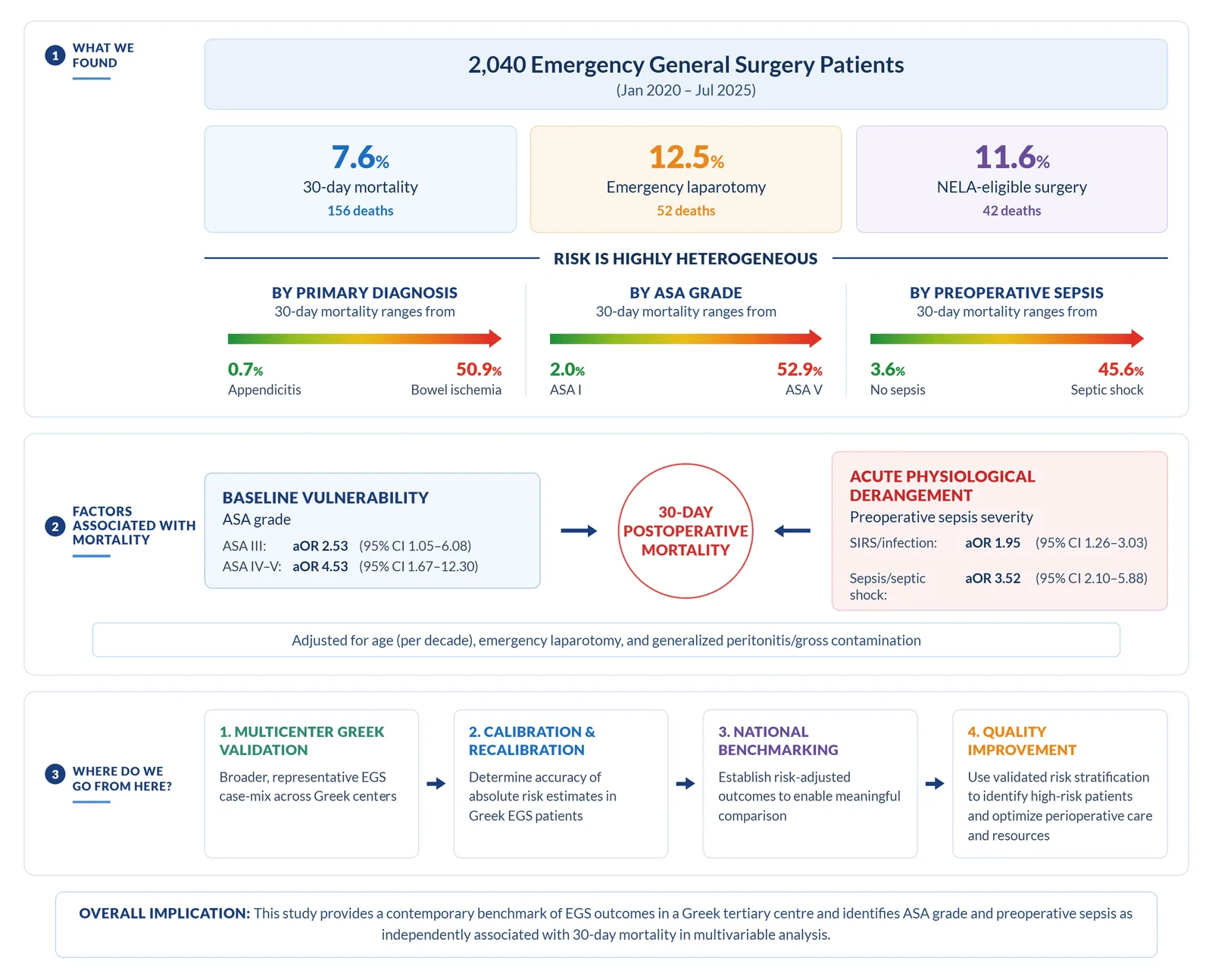
Key findings and future perspectives for emergency general surgery in Greece Image Credits: Alexis Terras, Nikolaos Machairas using Canva (Canva Pty Ltd., Sydney, Australia)

## Conclusions

In this single-center Greek cohort, 30-day mortality after emergency general surgery was 7.6% (156/2040) overall, rising to 12.5% (52/415) among patients undergoing emergency laparotomy and 11.6% (42/361) among NELA-eligible emergency laparotomies, with a clear gradient across diagnosis groups, ASA grade, and preoperative sepsis category. ASA grade and preoperative sepsis severity were independently associated with death in the multivariable model, broadly consistent with predictors identified in an earlier cohort. These estimates are, however, conditional on the model specification and should be interpreted accordingly. Sensitivity analyses showed that alternative specifications yield different sets of independently significant covariates: entering mesenteric ischemia - the single most lethal presentation in this cohort (50.9% mortality) - produced an independent association and attenuated the adjusted estimates for both ASA IV-V and sepsis/septic shock, while replacing ASA grade with comorbidity and frailty measures rendered generalized peritonitis significant. Variables not retained in the primary model should therefore not be regarded as lacking independent prognostic value, and residual confounding, particularly by diagnostic category, cannot be excluded. Established risk-prediction scores showed moderate discrimination on exploratory analysis; calibration was not assessed in this study and requires dedicated evaluation, particularly given the calibration problems recently reported for these same tools in a larger Greek multicenter validation. These findings extend the emerging body of Greek emergency-surgery outcomes evidence beyond emergency laparotomy to the broader operatively managed EGS cohort, providing a local outcome benchmark and supporting formal multicenter validation of prediction tools in Greek EGS practice.
